# Methotrexate, Cyclosporine A, and Biologics Protect against Atherosclerosis in Rheumatoid Arthritis

**DOI:** 10.1155/2015/759610

**Published:** 2015-05-18

**Authors:** Bartłomiej Kisiel, Robert Kruszewski, Aleksandra Juszkiewicz, Anna Raczkiewicz, Artur Bachta, Małgorzata Tłustochowicz, Jadwiga Staniszewska-Varga, Krzysztof Kłos, Krzysztof Duda, Romana Bogusławska-Walecka, Rafał Płoski, Witold Tłustochowicz

**Affiliations:** ^1^Department of Internal Diseases and Rheumatology, Military Institute of Medicine, Ulica Szaserów 128, 04-141 Warszawa, Poland; ^2^Department of Infectious Diseases and Allergology, Military Institute of Medicine, Ulica Szaserów 128, 04-141 Warszawa, Poland; ^3^Department of Radiology, Military Institute of Medicine, Ulica Szaserów 128, 04-141 Warszawa, Poland; ^4^Department of Medical Genetics, Medical University in Warsaw, Ulica Pawińskiego 3c, 02-106 Warszawa, Poland

## Abstract

*Introduction*. The risk of cardiovascular disease is increased in rheumatoid arthritis (RA). A meta-analysis showed increased intima media thickness (IMT) in RA. It has been shown that disease modifying antirheumatic drugs (DMARDs) may influence the progression of atherosclerosis. However, it was suggested that biologics may be more efficient than other DMARDs (including methotrexate—MTX) in protecting against atherosclerosis. *Objectives*. The aim of this study was to assess the influence of different RA characteristics and treatment regimens on IMT and atherosclerotic plaques. *Patients and Methods*. 317 RA patients and 111 controls were included in the study. IMT was measured in carotid (CIMT) and femoral (FIMT) arteries. Arteries were screened for the presence of plaques. *Results*. CIMT, FIMT, and prevalence of plaques were lower in patients treated with methotrexate (MTX) ≥ 20 mg/wk, cyclosporine (CsA), or biologics than in patients treated with lower doses of MTX and other disease modifying antirheumatic drugs. No differences in IMT between patients treated with MTX ≥ 20 mg/wk, biologics, or CsA were found. *Conclusions*. We found a beneficial effect of MTX ≥ 20 mg/wk, biologics, and CsA on atherosclerosis. We do not confirm a stronger influence of biologics on IMT compared with therapeutic doses of MTX.

## 1. Introduction

Cardiovascular disease (CVD) morbidity and mortality rates are increased in RA patients compared to general population [1]. It is estimated that CVD in RA leads to an excess 35–50% of the mortality rate in comparison to general population and reduces life expectancy by 5–10 years [2, 3]. The pathogenesis of accelerated atherosclerosis in RA is postulated to be multifactorial. It has been shown that traditional CV risk factors like hypertension, diabetes, and hyperlipidemia contribute to the development of atherosclerosis in RA. However, the excess CV risk in RA persists after adjustment for established CV risk factors; thus RA is considered as an independent CV risk factor [4–7].

Several noninvasive diagnostic tools such as assessment of endothelial function, measurement of carotid intima media thickness (CIMT), and assessment of coronary artery calcification score may be used to detect subclinical atherosclerosis. A meta-analysis showed that CIMT predicts future vascular events in healthy individuals [8]. Some studies suggest that carotid and femoral arteries respond differently to CV risk factors and that inclusion of femoral artery IMT measurements would add information to that provided by the common carotid artery [9].

Studies in RA patients showed a decrease in flow mediated dilatation and an increase in augmentation index and pulse wave velocity, which suggests endothelial dysfunction [10–13]. Several studies have also shown increased CIMT and formation of plaques within the carotid artery in RA [10, 13, 14]. Increased CIMT in RA patients was also confirmed by a meta-analysis [15].

Several studies have focused on the influence of classical CV risk factors and disease-related factors on atherosclerosis in RA. Smoking is one of the most important CV risk factors but it is also known as a risk factor for the development of RA [16]. Thus smoking is frequently seen in RA patients and may provide a potential bias in studies on RA and CVD [17]. The relation between body mass index (BMI), RA, and CVD is also complex. On the one hand obesity is associated with CV morbidity and mortality [18, 19]. On the other hand, CV mortality is also increased in RA patients with a BMI below 20 kg/m^2^ [20]. A possible explanation for this excess CV risk is that low BMI may indicate the presence of rheumatoid cachexia [17]. Hypertension, another classical CV risk factor, is common in RA and was shown to be associated with atherosclerosis [17, 21]. The relation between lipid profile and CVD in RA is complex. The active inflammatory state of RA may lower levels of circulating lipids (i.e., total, LDL and HDL cholesterol, and triglycerides) [22]. However, these changes in lipid profile are associated with increased CV risk. This phenomenon is called lipid paradox and is probably due not only to low levels of HDL cholesterol but also to structural and functional changes of HDL [23, 24]. Treatment with disease modifying antirheumatic drugs (DMARDs) was shown to increase lipid levels. However, it is believed that these changes may reflect normalization of the lipid profile. Thus, the interpretation of lipid levels for predicting CV risk in RA patients should be cautious [25].

Among RA-related factors influencing atherosclerosis inflammation seems to play a major role. Wållberg-Jonsson et al. found that high disease activity was associated with increased risk of CV event and decreased life span [26]. A study by Maradit-Kremers et al. showed that markers of systemic inflammation confer additional risk for CV death among patients with RA [2]. Innala et al. reported that erythrocyte sedimentation rate (ESR) and cumulative disease activity (defined as area under the curve DAS28) increased a hazard rate for a new CV event [27]. Several studies also found the association between markers of inflammation and subclinical atherosclerosis. ESR and C-reactive protein (CRP) were found to be associated with CIMT, atherosclerotic plaques, arterial stiffness, flow-mediated dilatation (FMD), and glyceryl trinitrate-mediated dilatation (GMD) [5, 28–31]. On the other hand some studies failed to find the association between cumulative inflammation and markers of atherosclerosis [32–34]. Other RA-related factors which may predict progression of atherosclerosis are rheumatoid factor (RF), anti-citrullinated peptide antibodies (ACPA), disease duration, and radiological damage index [5, 34, 35].

It is well established that DMARDs therapy decreases CV morbidity and mortality. However, most studies focused on methotrexate (MTX) and biologics, while little is known about other DMARDs. Moreover, only few reports compared influence of different DMARDs on subclinical atherosclerosis. A prospective study by Choi et al. showed that treatment with methotrexate reduces CV mortality in RA patients [36]. A systematic review confirmed that the use of MTX decreases CV morbidity and mortality [37]. Few small studies found a beneficial effect of combined DMARDs therapy (MTX, hydroxychloroquine, and sulfasalazine) on CIMT, FMD, and GMD [31, 38, 39]. Several studies showed a beneficial effect of anti-TNF-*α* therapy on subclinical atherosclerosis [40–42] and systematic review by Westlake et al. confirmed that anti-TNF-*α* therapy reduces the likelihood of CVD in RA [43]. Interestingly, some studies suggest that biologics may be more efficient than MTX in protecting against atherosclerosis in RA. A study by Giles et al. showed slower progression of CIMT in patients treated with anti-TNF-*α* compared to those not receiving treatment; such association was not observed with other DMARDs [44]. Similarly, an analysis of large RA registry (CORRONA) showed reduction of CV events risk in patients treated with anti-TNF-*α* compared with patients treated with MTX and other nonbiological DMARDs [45].

The aim of this study was to assess the influence of different RA characteristics and treatment regimens on CIMT, FIMT, and atherosclerotic plaques.

## 2. Materials and Methods

The study was approved by the local ethical committee. All participants signed an informed consent form.

### 2.1. Patients

317 RA patients fulfilling the 1987 ACR criteria were recruited. Exclusion criteria comprised diabetes mellitus, coronary artery disease, and history of stroke. A complete history, physical examination, and laboratory evaluation were performed and recorded in a standard protocol (Table 1). All DMARDs ever used were recorded unless treatment duration was <3 months. Patients were divided into 2 groups: continuously (cDMARDs) and discontinuously (dDMARDs) treated with DMARDs (treatment with DMARDS ≥ 90 and <90% of RA duration, resp.). Hands and feet X-rays were performed in most patients. RA activity was assessed with DAS28. Framingham 10-year risk score (FSS) was used to estimate general CV risk related to classical risk factors [46].

### 2.2. Controls

111 age- and sex-matched healthy individuals were included in the control group. Clinical and laboratory data are summarized in Table 1.

### 2.3. Ultrasonography

IMT was measured on the far wall of the common carotid and superficial femoral arteries. Atherosclerotic plaque was defined as a focal structure that encroaches into the arterial lumen of at least 0.5 mm or 50% of the surrounding IMT or demonstrates a thickness of ≥1.5 mm as measured from the media-adventitia interface to the intima-arterial lumen interface. CIMT and FIMT were defined as a mean value of 6 measurements (CIMT: 1, 2, and 3 cm proximal to the bifurcation bilaterally; FIMT: 1, 2, and 3 cm distal to the bifurcation bilaterally). Common carotid and superficial femoral arteries were investigated for the presence of plaques.

### 2.4. Statistical Analysis

All statistical tests were performed with STATISTICA 10.0 (StatSoft). Results are reported as mean (SD) for continuous variables and *n* (%) for categorical variables. According to data distribution, a parametric (*t*-test) or nonparametric (*U* Mann-Whitney) test was used. Categorical variables were compared with chi square exact test. A *P* value < 0.05 was considered significant.

## 3. Results and Discussion

### 3.1. Results

Patients and controls were age- and sex-matched. The percentage of ever-smokers, LDL, and total cholesterol concentrations and BMI were higher in controls than in RA (Table 1). However, total CV risk calculated with FSS was similar in both groups. CIMT and FIMT were higher in RA but only the difference in FIMT was significant. Atherosclerotic plaques were more prevalent in RA.

The presence of plaques in RA was positively correlated with ESR, creatinine concentration, FSS, and presence of rheumatoid factor (RF) (Table 2). Analysis for associations between plaques and treatment with DMARDs showed a significant negative correlation between presence of plaques and treatment with methotrexate (MTX), cyclosporine A (CsA), and biologics. Plaques were insignificantly more prevalent in dDMARDs group than in cDMARDs group.

We found a positive correlation between CIMT, FIMT, and FSS (*r* = 0.488, *P* < 0.001, and *r* = 0.434, *P* < 0.001, resp.), ESR (*r* = 0.132, *P* = 0.018, and *r* = 0.199, *P* < 0.001), and creatinine concentration (*r* = 0.2, *P* < 0.001, and *r* = 0.212, *P* < 0.001). However, after adjustment for age, associations with creatinine became insignificant. No significant associations were found between CIMT, FIMT, and RA duration, CRP concentration, and DAS28 (data not shown).

CIMT and FIMT were significantly lower in cDMARDs group compared with dDMARDs group (Table 3). The association remained significant after adjustment for classical CV risk factors. The use of MTX was associated with lower FIMT. Comparison of different doses of MTX revealed significantly lower CIMT and FIMT in patients treated with doses ≥20 mg/wk; correlation remained significant after adjustment for classical CV risk factors. CIMT was also significantly lower in patients treated with CsA and biologics. A similar correlation was observed between CsA, biologics, and FIMT but it became insignificant after correction for CV risk factors. We did not find significant differences in CIMT and FIMT in pairwise comparisons between patients treated with MTX ≥ 20 mg/wk, biologics, or CsA (further named MTX20(+)/CsA(+)/biologics(+) group); a comparison of this group with patients treated with different DMARDs/lower doses of MTX (further named MTX20(−)/CsA(−)/biologics(−) group) revealed a robust difference in CIMT (0.104 mm, *P* = 1 × 10^−6^) and FIMT (0.081 mm, *P* = 5 × 10^−5^). Interestingly, RA activity (measured by DAS28) was similar in both groups: 4.64 (1.54) versus 4.83 (1.57), *P* = 0.3. No significant differences in classical CV risk factors were found between patients treated with MTX ≥ 20 mg/wk, biologics, or CsA. CIMT in MTX20(+)/CsA(+)/biologics(+) was comparable to controls. FIMT was slightly higher in MTX20(+)/CsA(+)/biologics(+) group than in controls. No correlations were found between CIMT and FIMT and presence of RF, ACPA, and bone erosions.

### 3.2. Discussion

RA patients are at higher risk of CVD than an age-matched general population. It is estimated that CV risk in RA is increased to a similar magnitude to that seen in type 2 diabetes [47]. Studies assessing IMT in RA showed conflicting results but two meta-analyses confirmed increased IMT in RA [15, 48]. We also observed increased IMT in carotid and femoral arteries in RA patients, but only the difference in femoral arteries was significant. Atherosclerotic plaques were more frequently found in RA than in controls.

We found a strong correlation between FSS and IMT and presence of plaques. This finding underlines the role of classical CV risk factors in pathogenesis of atherosclerosis in RA.

CIMT, FIMT, and presence of plaques were associated with ESR. However, no correlation was found between atherosclerosis markers and DAS28. It may be explained by the fact that DAS28 comprises two parameters which are not completely objective: VAS and tender joints count. Moreover, swollen joints count is related to the local inflammation (synovitis), while progression of atherosclerosis in RA is thought to be due to systemic inflammation. Thus ESR, as a more objective parameter and a marker of systemic inflammation, may be a better predictor of increased risk of atherosclerosis in RA. In this context lack of association with CRP is intriguing. CRP was found to be a powerful predictor of cardiovascular disease in general population [49]. The absence of association in our study may be explained by the use of conventional CRP assay, since the involvement of CRP in atherosclerosis has been demonstrated by high sensitivity CRP assay. It must be also emphasized that RA is a disease characterized by periods of exacerbations and remissions. Thus, a single measure of disease activity may not reflect intensity of disease in a longer period of time. Indeed, several studies suggest that assessment of the cumulative inflammation for the whole duration of RA may be a better predictor of atherosclerosis [34].

Creatinine concentration was significantly higher in patients with plaques and correlated positively with CIMT and FIMT. However, after adjustment for age these associations became insignificant (data not presented). It is not surprising as a concentration of creatinine increases with age and age is a strong risk factor for atherosclerosis.

CIMT, FIMT, and prevalence of plaques were lower in patients treated with MTX ≥ 20 mg/wk, CsA, and biologics. This effect seems to be independent of disease activity as DAS28 was similar in MTX20(+)/CsA(+)/biologics(+) and MTX20(−)/CsA(−)/biologics(−) groups. Analysis of different combinations of DMARDs did not reveal any significant correlations (data not shown). There is a lot of evidence supporting a beneficial effect of biologics on atherosclerosis, while data concerning MTX are conflicting. Several studies showed a beneficial influence of anti-TNF-*α* therapy on subclinical atherosclerosis [40–42]. A protective effect of anti-TNF-*α* therapy was also confirmed in meta-analyses [43, 50]. A study based on data from British Society for Rheumatology Biologics Register showed no overall difference in the risk of myocardial infarction between patients treated with anti-TNF-*α* and nonbiologic DMARDs; however, the authors reported a reduced risk of myocardial infarction in TNF-*α*-responders [51]. Giles et al. observed slower CIMT progression in patients treated with anti-TNF-*α*, but not in users of other RA treatments [44]. Analysis of CORRONA registry showed reduction of CV events risk in patients treated with anti-TNF-*α* compared with patients treated with MTX and other nonbiological DMARDs [45]. On the other hand a systematic review by Westlake et al. found that use of MTX is associated with reduced risk of CV events [37]. Our results suggest that the observed discrepancy in the literature may be due to different doses of MTX. For last three decades doses of MTX used in RA have increased from 5–7.5 mg/wk to 30 mg/wk. A study by Giles et al. enrolled patients between 2004 and 2006 and enrolment to CORRONA registry took place between 2001 and 2006; data concerning average dose of MTX in these studies is missing in the publications; however, we may speculate that it was below 20 mg/wk. We observed a significant difference in CIMT and FIMT between patients treated with MTX ≥ 20 mg/wk and <20 mg/wk; it should be emphasized that the difference remained significant after exclusion of patients treated with other drugs influencing IMT (i.e., CsA and biologics). We did not observe significant differences in IMT between patients treated with MTX ≥ 20 mg/wk (but never using biologics) and patients treated with biologics (but never using MTX ≥ 20 mg/wk). It suggests that the impact of MTX ≥ 20 mg/wk on IMT was comparable to that of biologics.

A beneficial influence of CsA on atherosclerosis in RA is a novel finding. Few studies reported a protective effect of CsA on IMT in lupus patients [52]. Surprisingly, other synthetic DMARDs recommended in RA (leflunomide, sulphasalazine) showed no effect on IMT and presence of plaques.

The differences in CIMT and FIMT between MTX20(+)/CsA(+)/biologics(+) and MTX20(−)/CsA(−)/biologics(−) groups were robust (0.104 mm, *P* = 1 × 10^−6^, and 0.081 mm, *P* = 5 × 10^−5^). A large study in general population found that an absolute carotid IMT difference of 0.1 mm is associated with a 10–15% higher risk of myocardial infarction and 13–18% higher risk of stroke [53]. Thus, the observed effect of MTX, biologics, and CsA seems to be important.

Another factor influencing the atherosclerosis status is regularity of treatment. Patients treated continuously with DMARDS had a lower CIMT and FIMT. This finding is not surprising as good RA control is a widely accepted predictor of slower atherosclerosis progression.

Lekakis et al. suggested that combined assessment of carotid and femoral IMT might provide additional information compared with analysis of carotid IMT only [9]. Our results suggest that this hypothesis may not be applicable to RA population.

## 4. Conclusions

In conclusion, we found a beneficial effect of MTX ≥ 20 mg/wk, biologics, and CsA on atherosclerosis. We do not confirm a stronger influence of biologics on IMT compared with MTX (in doses ≥20 mg/wk).

## Figures and Tables

**Table 1:** Study and control group characteristics.

	RA (*n* = 317)	Controls (*n* = 111)	*P* value
Age, years	57.61 (12.62)	55.50 (9.37)	0.1
Males	58 (18.30%)	22 (19.81%)	0.7
Ever-smokers	140 (44.16%)	62 (55.86%)	0.04
Pack-years	9.66 (16.04)	13.13 (18.20)	0.06
BMI, kg/m^2^	25.54 (4.37)	27.46 (4.69)	0.0001
Hypertension	137 (43.22%)	38 (34.23%)	0.1
Creatinine, mg/dL	0.73 (0.27)^‡^	0.65 (0.07)^▼^	0.3
ESR, mm/h	31.77 (23.64)	10.63 (9.19)	<1 × 10^−6^
CRP, mg/dL	2.15 (3.2)	0.43 (0.641)	<1 × 10^−6^
Total cholesterol, mg/dL	203.8 (41.3)	215.8 (44.3)	0.01
LDL cholesterol, mg/dL	115.7 (34.3)^†^	127.4 (40.5)	0.004
HDL cholesterol, mg/dL	63.2 (19.7)^†^	66.5 (19)	0.1
Triglycerides, mg/dL	128.6 (60.5)^†^	116.2 (56.8)	0.06
Framingham 10-year risk score	7.17 (5.4)^†^	7.86 (6.32)	0.3
Presence of atherosclerotic plaques in carotid and/or femoral arteries	74 (23.34%)	14 (12.61%)	0.015
CIMT, mm	0.718 (0.181)	0.682 (0.167)	0.07
FIMT, mm	0.516 (0.168)	0.457 (0.099)	0.0005
Disease duration, years	10.74 (8.98)		
Methotrexate ever	303 (95.58%)		
Sulphasalazine ever	148 (46.69%)		
Hydroxychloroquine or chloroquine ever	98 (30.91%)		
Gold salts ever	46 (14.51%)		
Azathioprine ever	18 (5.68%)		
Cyclophosphamide ever	7 (2.21%)		
Cyclosporine A ever	77 (24.29%)		
Leflunomide ever	123 (38.8%)		
Biologic agents ever	61 (19.24)		
Infliximab ever	24 (7.57%)		
Adalimumab ever	12 (3.78%)		
Etanercept ever	39 (12.3%)		
Rituximab ever	13 (4.1%)		
Continuous treatment with DMARDs	141 (45.19%)^¶^		
RF positivity	217 (70.68%)^#^		
ACPA positivity	211 (77.29%)^§^		
DAS28	4.7 (1.55)		
Presence of erosions in hand and/or feet X-ray	176 (70.97%)^▲^		

Data is presented as mean (standard deviation) for continuous variables and number (percentage) for categorical variables. ^¶^Data available for 312 patients. ^#^Data available for 307 patients. ^§^Data available for 273 patients. ^‡^Data available for 286 patients. ^†^Data available for 302 patients. ^▲^Data available for 248 patients. ^▼^Data available for 102 patients.

**Table 2:** Associations between presence of atherosclerotic plaques and clinical, laboratory, and radiological characteristics and use of different DMARDs.

	Presence of atherosclerotic plaques in carotid and/or femoral arteries (*n* = 74)	Lack of atherosclerotic plaques in carotid and femoral arteries (*n* = 242)	*P*
RA duration, years	10.54 (9.41)	10.76 (8.86)	0.8
ESR, mm/h	31.17 (24.39)	22.49 (21.4)	0.0008
CRP, mg/dL	1.99 (2.9)	1.42 (2.66)	0.07
Creatinine, mg/dL	0.81 (0.3)	0.7 (0.21)	0.0007
DAS28	4.63 (1.51)	4.73 (1.57)	0.6
Framingham 10-year risk score	10.1 (6.22)	6.01 (5.16)	<1 × 10^−6^
Methotrexate ever	65 (87.84%)	237 (97.93%)	0.0002
Sulphasalazine ever	30 (40.54%)	117 (48.35%)	0.2
Hydroxychloroquine or chloroquine ever	22 (29.72%)	76 (31.4%)	0.8
Gold salts ever	8 (10.81%)	38 (15.7%)	0.3
Azathioprine ever	5 (6.76%)	13 (5.37%)	0.7
Cyclophosphamide ever	2 (2.7%)	5 (2.07%)	0.7
Cyclosporine A ever	10 (13.51%)	67 (27.69%)	0.01
Leflunomide ever	28 (37.84%)	95 (39.26%)	0.8
Biologic agents ever	5 (6.76)	56 (23.14%)	0.002
Infliximab ever	1 (1.35%)	23 (9.5%)	0.02
Adalimumab ever	0 (0%)	12 (4.96%)	0.0503
Etanercept ever	2 (2.7%)	37 (15.29%)	0.004
Rituximab ever	0 (0%)	13 (5.37%)	0.04
Continuous treatment with DMARDs	27 (36.49%)	114 (47.11%)	0.1
RF positivity	59 (79.73%)	158 (67.81)^▲^	0.0497
ACPA positivity	49 (76.56%)^#^	162 (77.51%)^†^	0.9
Presence of erosions in hand and/or feet X-ray	42 (68.58%)^‡^	134 (71.66%)^§^	0.8

Data is presented as mean (standard deviation) for continuous variables and number (percentage) for categorical variables. ^#^Data available for 64 patients. ^‡^Data available for 61 patients. ^▲^Data available for 233 patients. ^†^Data available for 209 patients. ^§^Data available for 187 patients.

**Table 3:** Associations between IMT and use of different DMARDs, treatment regimen, presence of RF, ACPA, and erosions.

	Significant differences in classical CV risk factors (i.e., age, BMI, smoking, hypertension, lipid profile, and FSS) between groups	CIMT			FIMT		
		CIMT, mm	*P*	*P*_adj_	FIMT, mm	*P*	*P*_adj_
MTX(+) versus MTX(−)	—	0.716 (0.178) versus 0.784 (0.235)	0.2		0.511 (0.159) versus 0.628 (0.293)	0.01	0.03

MTX ≥ 20 mg/wk versus MTX < 20 mg/wk	MTX ≥ 20 mg/wk group was younger (55.77 yrs versus 59.85 yrs) and had lower FSS (6.57 versus 7.92)	0.687 (0.171) versus 0.758 (0.186)	0.0005	0.009	0.492 (0.126) versus 0.546 (0.206)	0.004	0.04

Sulphasalazine(+) versus sulphasalazine(−)	—	0.707 (0.168) versus 0.731 (0.192)	0.2		0.505 (0.138) versus 0.526 (0.192)	0.3	

Hydroxychloroquine/chloroquine(+) versus hydroxychloroquine/chloroquine(−)	—	0.703 (0.181) versus 0.727 (0.181)	0.3		0.517 (0.163) versus 0.515 (0.181)	0.9	

Gold salts(+) versus gold salts(−)	—	0.682 (0.139) versus 0.726 (0.187)	0.1		0.494 (0.166) versus 0.520 (0.169)	0.3	

Azathioprine(+) versus azathioprine(−)	—	0.731 (0.216) versus 0.719 (0.179)	0.8		0.518 (0.110) versus 0.516 (0.172)	0.96	

Cyclophosphamide(+) versus cyclophosphamide(−)	—	0.745 (0.205) versus 0.719 (0.181)	0.7		0.669 (0.427) versus 0.512 (0.158)	0.01	0.2

Cyclosporine(+) versus cyclosporine(−)	CsA(+) group was younger (53.03 yrs versus 58.46 yrs) and had lower FSS (5.91 versus 7.59), and hypertension was less prevalent in this group (32.47% versus 46.44%)	0.665 (0.165) versus 0.737 (0.183)	0.002	0.03	0.471 (0.095) versus 0.530 (0.184)	0.007	0.06

Leflunomide(+) versus leflunomide(−)	—	0.719 (0.174) versus 0.720 (0.186)	0.96		0.514 (0.161) versus 0.517 (0.174)	0.9	

Biologic agents(+) versus biologic agents(−)	Biologic agents(+) group was younger (53.24 yrs versus 58.31 yrs)	0.663 (0.165) versus 0.733 (0.182)	0.006	0.04	0.477 (0.124) versus 0.525 (0.177)	0.04	0.2

MTX20(+)/CsA(+)/biologics(+) versus MTX20(−)/CsA(−)/biologics(−)	MTX20(+)/CsA(+)/biologics(+) group was younger (55.47 yrs versus 61.59 yrs) and had lower FSS (6.38 versus 8.82)	0.684 (0.169) versus 0.788 (0.186)	1 × 10^−6^	2 × 10^−5^	0.489 (0.123) versus 0.570 (0.227)	5 × 10^−5^	6 × 10^−4^

MTX20(+)/CsA(+)/biologics(+) versus controls	—	0.684 (0.169) versus 0.682 (0.167)	0.94		0.489 (0.123) versus 0.457 (0.099)	0.02	0.02

MTX20(+)/biologics(−) versus biologics(+)MTX20(−)	—	0.703 (0.173) versus 0.708 (0.172)	0.9		0.493 (0.128) versus 0.458 (0.123)	0.2	

Biologics(+)/CsA(−) versus CsA(+)/biologics(−)	—	0.655 (0.181) versus 0.662 (0.176)	0.9		0.476 (0.155) versus 0.467 (0.102)	0.3	

CsA(+)/MTX20(−) versus MTX20(+)/CsA(−)	—	0.661 (0.144) versus 0.696 (0.168)	0.3		0.473 (0.076) versus 0.500 (0.134)	0.3	

MTX ≥ 20/biologics(−)/CsA(−) versus MTX < 20/biologics(−)/CsA(−)	MTX ≥ 20/biologics(−)/CsA(−) group was younger (56.41 yrs versus 60.39 yrs) and had lower FSS (6.71 versus 8.01)	0.708 (0.164) versus 0.791 (0.188)	0.0009	0.008	0.502 (0.130) versus 0.577 (0.227)	0.004	0.02

cDMARDs versus dDMARDs	cDMARDs group was younger (54.53 yrs versus 59.62 yrs)	0.683 (0.177) versus 0.746 (0.182)	0.002	0.02	0.485 (0.114) versus 0.544 (0.200)	0.002	0.01

RF(+) versus RF(−)	—	0.733 (0.189) versus 0.690 (0.160)	0.06		0.524 (0.164) versus 0.506 (0.182)	0.4	

ACPA(+) versus ACPA(−)	—	0.720 (0.180) versus 0.702 (0.202)	0.5		0.525 (0.171) versus 0.480 (0.148)	0.06	

Bone erosions(+) versus bone erosions(−)	—	0.730 (0.190) versus 0.721 (0.174)	0.8		0.530 (0.184) versus 0.501 (0.121)	0.2	

Data is presented as mean (standard deviation). Biologics(+)/MTX20(−): patients treated with biologics but never treated with MTX ≥ 20 mg/wk. Biologics(+)/CsA(−): patients treated with biologics but never treated with CsA. CsA(+)/MTX20(−): patients treated with CsA but never treated with MTX ≥ 20 mg/wk. CsA(+)/biologics(−): patients treated with CsA but never treated with biologics. MTX20(+)/biologics(−): patients treated with MTX ≥ 20 mg/wk but never treated with biologics. MTX20(+)/CsA(−): patients treated with MTX ≥ 20 mg/wk but never treated with CsA. MTX20(+)/CsA(+)/biologics(+): patients treated with MTX ≥ 20 mg/wk and/or CsA and/or biologic agents. MTX20(−)/CsA(−)/biologics(−): patients never treated with CsA, biologic agents, and MTX ≥ 20 mg/wk. MTX ≥ 20/biologics(−)/CsA(−): patients treated with MTX ≥ 20 mg/wk but never treated with biologics and CsA. MTX < 20/biologics(−)/CsA(−): patients treated with MTX < 20 mg/wk but never treated with biologics and CsA. *P*_adj_: *P* value for analysis adjusted for classical CVD risk factors.
